# Changes in Emergency Department Performance during Strike of Junior Physicians in Korea

**DOI:** 10.1155/2021/1786728

**Published:** 2021-07-08

**Authors:** Jeongyong Sim, Yuri Choi, Jinwoo Jeong

**Affiliations:** ^1^Department of Emergency Medicine, Dong-A University Hospital, Busan, Republic of Korea; ^2^Department of Emergency Medicine, Dong-A University College of Medicine, Busan, Republic of Korea

## Abstract

**Objective:**

A nationwide strike that took place from August 21 to September 7, 2020, which was led by young doctors represented by residents and interns, resulted in shortages of manpower at almost all university and training hospitals. This study aimed to identify differences in the process and outcomes of emergency department (ED) patient care by comparing the performance over about 2 weeks of the strike with that during the usual ED operations.

**Methods:**

This retrospective observational study evaluated ED flow and performance during the junior doctors' strike and compared it with the usual period in a single tertiary-care academic hospital. The outcome variables were defined as ED length of stay, crude mortality, and hospital mortality and adjusted for demographic and clinical parameters. The effect of the doctors' strike on hospital mortality adjusted for demographic and clinical variables was investigated using logistic regression.

**Results:**

A total of 1,121 and 1,496 patients visited the ED during the strike and control periods (both 17 days), respectively. The care usually provided by four or six physicians, including one specialist, was replaced with that by one or two specialists at any one time. During the trainee doctors' strike, EM specialists managed patients with fewer consultations. However, the proportion of patients who underwent laboratory and radiologic tests did not change significantly. The median ED length of stay significantly decreased from 359 minutes (interquartile range, IQR: 147–391) in the control period to 326 minutes (IQR: 123–318) during the strike period (*P* < 0.001). The doctors' strike was not found to have a significant effect on mortality after adjustments with other variables.

**Conclusion:**

During the junior doctors' strike in 2020 in Korea, EM specialists efficiently managed the care of emergency patients with higher levels of acuity without compromising the survival rate, through fewer consultations and faster disposition.

## 1. Introduction

Historically, doctors have gone on strike to express their opinions in the same manner as members of other professions [1–5]. In the UK, residents engaged in a strike against new work contracts from 2014 to 2016 [6]. Israeli physicians went on strike to express their concerns about salaries and working conditions in 2011 [5]. There have been several doctors' strikes in South Korea, the largest of which was a response to the government's new medical policy in 2000 [7, 8]. The strike began with doctors in clinics and community hospitals, and participation then expanded to doctors from universities including residents, attending physicians, and faculties with varying austerity for a total of five months [7–10].

In 2020, a nationwide strike was launched to protest the plan regarding a medical school for public health in Korea. It was led by young doctors represented by residents and interns and resulted in shortages of manpower at almost all the university hospitals and training hospitals [11], with medical students participating by boycotting class activities. While most clinics and community hospitals did not take part in the strike, most resident physicians, who are key personnel in universities and training hospitals, were absent from essential services such as emergency departments (EDs), intensive care units, operating theaters, and hemodialysis units throughout the strike. Owing to their absenteeism, for approximately 2 weeks, the faculty and attending physicians were the only doctors providing elective and emergency care in academic hospitals. Concerns about possible decreased quality in medical care and an increase in mortality arose, considering the preexisting difficulties posed by the outbreak of the coronavirus disease (COVID-19).

Large-scale strikes of doctors have been the focus of research, as they can be considered a unique type of medical disaster caused by supply shortages when disasters are defined as a disruption of the functioning of a society that exceeds the affected society's ability to cope using its own resources [12]. Strikes of trainee physicians in university hospitals can be seen as a “natural experiment” that can elucidate the differences in the treatment processes conducted by residents and specialists [13]. Therefore, the authors tried to compare the ED performance over about two weeks of the strike with that of usual ED operations to identify differences in the process and outcome of ED patient care caused by the strike.

## 2. Materials and Methods

### 2.1. Study Design

This study was a retrospective observational study evaluating ED flow and performance during the doctors' strike compared with the usual period in an academic ED in a metropolitan city in Korea. The study was approved by the Institutional Review Board of the study hospital and informed consent was waived due to the retrospective nature of the study (DAUHIRB-21-032). The funding organization was not involved in the study's reporting process.

### 2.2. Study Setting and Population

The setting of this study was a tertiary-care academic hospital with 997 inpatient beds that is designated as a regional emergency medical center. The ED is visited by approximately 32,000 patients annually. Junior doctors commenced a strike from August 21^st^ to September 7^th^, 2020, to protest against the government's policies regarding medical human resources. Likewise, all the residents and interns in the study hospital participated in the strike in South Korea.

**Figure 1:**
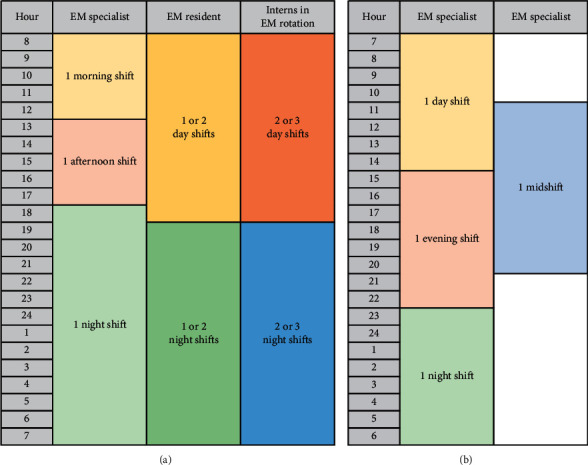
Timetable presenting the physician workforce in the study ED during the usual period and the junior doctor's strike period. ED: emergency department; EM: emergency medicine. (a) Usual physician workforce in the study ED. (b) ED physician workforce during the strike.

The ED was usually operated by a total of 17 physicians: 6 emergency medicine specialists, 5 EM residents, and 6 interns. Four to six physicians worked at the same time, with rotating shifts. However, since the start of the strike, only six specialists worked on three shifts, with one or two of them working at the same time. The ED physician workforce's timetables during the usual and strike periods are presented in Figure 1. The structure of human resources in other groups in the ED remained the same, composed of nurses, nursing assistants, emergency medical technicians (EMT), and administrative staff. The scope of the ED doctor's work included history taking and physical examinations, bedside procedures, prescriptions of laboratory and radiologic tests, request for consultations with other specialists, and the acquisition of informed consent.

The strike period was defined as August 21^st^, 2020 (Friday), at 8 a.m. to September 7^th^, 2020 (Monday), at the same time (17 days). The control period was defined to include 17 days shortly before the strike, that is, July 31^st^, 2020 (Friday), at 8 a.m. until August 17^th^, 2020 (Monday) at the same time, so that the same numbers of holidays and weekdays were included, and the effect of the COVID-19 pandemic and seasonal variation was similar.

### 2.3. Study Protocol

Data were extracted from the hospital information system. All ED visits during the strike and control periods were included because the variables used in the study were generally administrative and no data were missing. To estimate the effects of the doctors' strike on hospital mortality adjusted for demographic and clinical parameters, logistic regression was carried out. The following exclusion criteria were applied for regression modeling: cardiac arrest at presentation and missing or erroneous values with vital signs. Those younger than 15 years were also excluded because the reference range for vital signs was different from adults.

### 2.4. Measurements

Age; sex; event classification; transportation and route of the ED visit; the Korea Triage and Acuity Scale (KTAS) [14–16]; level of consciousness measured with the alert, verbal, pain, unresponsive (AVPU) scale; initial vital signs (systolic and diastolic blood pressure, heart rate, respiratory rate, body temperature, and pulse oxygen saturation at the time of ED arrival); the time of the ED visit, discharge, and admission; the contents of medication; and laboratory and diagnostic tests were retrieved from the hospital information system database. The KTAS is based on the Canadian Triage and Acuity Scale (CTAS) with some modifications considering the medical environment in Korea. Therefore, KTAS is very similar to CTAS and is composed of 5 levels, of which level 1 is the most emergent and level 5 is considered nonurgent [15, 17].

The outcome variables were defined as ED length of stay (EDLOS) and mortality. Mortality included mortality in the ED, hospital mortality after admission, and expected death after discharge.

### 2.5. Data Analysis

Continuous data were presented as medians and interquartile ranges (IQRs) and compared using Wilcoxon's rank-sum tests because the variables analyzed in the study, including age, appeared to have skewed distributions. Categorical data were summarized as frequencies (%) and compared using the chi-square test. The effect of the doctors' strike on hospital mortality adjusted for demographic and clinical variables was investigated using logistic regression. Because clinical parameters such as vital signs do not linearly correlate with severity or mortality, actual values were transformed into scores according to the table used for calculation of the National Early Warning Score before being incorporated into the logistic regression model [18–20]. The results of logistic regression are presented as odds ratio and 95% confidence intervals. *R* (version 4.0.3; *R* Foundation for Statistical Computing, Vienna, Austria, 2020) was used for statistical analyses. Statistical significance was set at *P* < 0.001.

## 3. Results

### 3.1. Characteristics of Study Subjects

**Figure 2:**
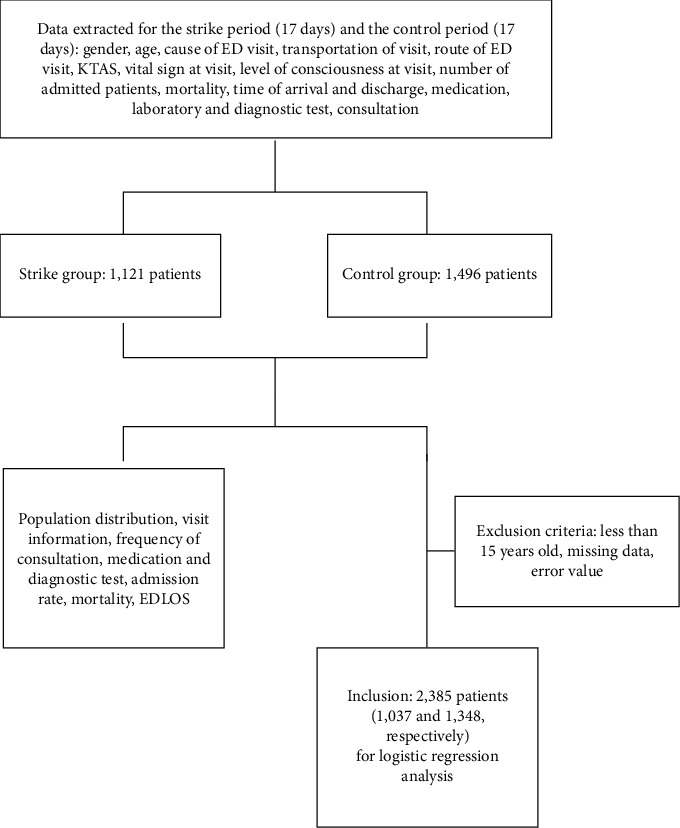
Flow diagram of included and excluded cases analyzed in the present study.

A total of 1,121 patients in the strike period and 1,496 patients in the control period visited the ED. After applying exclusion criteria, 1,037 cases in the strike group and 1,384 cases in the control group were finally included for analysis (Figure 2). There were no significant differences in age or sex between the study and control groups. In the strike periods, acuity measured with KTAS increased and the proportion of disease cases increased, while that of injury or poisoning cases decreased. The proportion of hospital admissions and crude mortality were not significantly different between the periods (Table 1).

### 3.2. Changes in Patient Care Activity

During the strike of doctors under training, EM specialists managed patients with fewer consultations. However, the proportion of patients who underwent laboratory and radiologic tests did not change significantly (Table 2, *P* < 0.001) (96.8% vs. 96.2% with laboratory tests and 92.1% vs. 92.8% with radiologic tests and strike vs. control period, respectively; *P*=0.476 and 0.495, respectively).

### 3.3. ED Length of Stay

The median ED length of stay (EDLOS) significantly decreased from 359 minutes (interquartile range, IQR: 147–391) in the control period to 326 minutes (IQR: 123–318) during the strike period (*P* < 0.001). The median time to disposition order also significantly decreased from 145.5 minutes (IQR: 83–234.75) to 115 minutes (IQR: 67.75–191) (*P* < 0.001).

### 3.4. Adjusted Mortality by Logistic Regression

After applying the exclusion criteria for logistic regression, 1,037 cases in the strike period and 1,348 cases in the control period were finally included in the severity-adjusted mortality analysis (Figure 1). After mutual adjustment, age, KTAS, consciousness, systolic blood pressure, and pulse oximetry were found to be significantly associated with mortality. Doctors' strike was not found to have a significant effect on mortality after adjustments with other variables (Table 3).

## 4. Discussion

This study revealed that ED visits decreased during the strike, EDLOS was reduced, and the quality of care assessed with severity-adjusted survival was maintained. ED crowding has been conceptualized into three interdependent components: input, throughput, and output [21]; similarly, we categorize the study results into three components for discussion: patient inflow, management process, and outcome.

### 4.1. Patient Inflow

There was an approximate 25% reduction in the total volume of ED visits during the strike, while the number of physicians working in the ED was reduced to one-third of the usual number. This result is contrary to those of previous studies that reported increased ED visits during the strike of Korean doctors in 2000. A multicenter study reported that, during the strike period, the number of patients was larger than normal on weekdays but was less than normal on consecutive holidays [9], while another study on the same strike period reported that the total of 475 patients during the strike period was significantly higher than the total of 234 patients during the nonstrike period [7]. The 2000 event was a nationwide strike in which doctors in clinics and nontraining hospitals also participated, and the EDs of academic hospitals functioned as a last resort. In contrast, the 2020 strike mainly involved trainee doctors and the functions of nontraining hospitals and clinics were preserved. It can thus be speculated that demands for emergency care were diverted to nontraining EDs in the 2020 strike. This study revealed that the number of higher acuity cases was relatively preserved, and the reduction in visits was greater in cases with KTAS 4 and 5. Therefore, it can be assumed that fewer acute patients diverted to the appropriate level of care.

### 4.2. Management Process

The EDLOS in the study period was significantly decreased, and the difference in median EDLOS was 33 minutes. Similarly, the median time to disposition orders decreased by 30 minutes. Therefore, the reduction in EDLOS can be attributed to a faster decision-making process rather than a decreased boarding time after disposition orders. Thornton et al. reported that all median time intervals within the total LOS in the ED were reduced during a junior doctor strike and suggested that the strike period patient flow was different from and better than the “standard” patient flow [22]. Salazar et al. also demonstrated that replacing residents with staff physicians resulted in shorter lengths of stays in the ED in urban teaching hospitals during a strike of resident physicians [23]. They explained that this major impact of the involvement of attending staff in the ED in the care of those patients with less severe conditions is consistent with a faster decision-making process and better clinical judgment by more experienced physicians [23]. The strike assessed in this study involved not only EM residents but also residents in all the other departments; therefore, specialist consultants participated in decision-making at an earlier stage than normal. We also found that the number of consultations per patient, which is considered to play a key role in delaying EDLOS [24, 25], was substantially reduced. It can be assumed that decisions made by EM specialists and the earlier involvement of specialists from other departments contributed to decreased EDLOS.

Although previous studies reported that direct care by emergency specialists reduced the frequency of radiologic or laboratory tests and increased the number of discharged patients who were only prescribed oral medication [9, 23], the study did not find any significant reduction in the proportion of patients prescribed with radiologic or laboratory tests. Detsky et al. suggested that the volume of tests performed in teaching hospitals is more likely related to the case mix and severity of illness of patients admitted to these institutions than to a pure “teaching effect” [13]. While a previous report by Choi et al. reported admission rates among ED patients of 10.2–27.6% [25], the admission rate of the ED in this study was much higher: 46.2% in the control period and 47.8% in the strike period. Reflected by the emergency admission rate, the severity of the study ED population was high; therefore, although the ED was operated by specialists without trainees, the necessity of diagnostic tests did not change and the frequency of tests was maintained.

### 4.3. Treatment Outcome

Although the physician workforce was reduced and the acuity of the patients increased, crude mortality did not increase during the strike. Previous studies have reported similar results; the nationwide mortality rate did not increase during the doctor's strike of 2000 [10]. In a university-affiliated teaching hospital in New Zealand, it was also reported that no difference was observed in ED mortality and 48-hour mortality during a resident's strike [26].

However, to properly compare the periods before and during the strike, mortality should be adjusted to demographic and clinical parameters. This study did not find a significant difference in mortality adjusted to other variables.

This study had several limitations. As it was a single-center observational study, it could not represent the different impacts suffered, and divergent strategies to confront the difficulties by other hospitals during the nationwide strike. And although survival in the study hospital did not increase, there is a possibility that nationwide or region-wide mortality may have changed because more patients were treated in community hospital EDs. A nationwide registry-based study would be required to elucidate the effect of junior doctors' strikes on the overall emergency medical system. While the quality of care measured by severity-adjusted survival was preserved, the ED operation during the strike was obviously difficult to maintain for an extended period because of the increased workload and fatigue imposed on EM specialists, factors that were not analyzed in this study. Further, patients' or their caregivers' satisfaction with ED care, which is a key aspect of the quality of ED care, was not considered. Additionally, there may have been other factors that influenced the ED performance. A reduction in elective surgeries and scheduled admissions might have led to fewer access blocks and pressure on the hospital's resources.

## 5. Conclusions

EM specialists managed to care for emergency patients with higher acuity without compromising the survival, with more efficiency through fewer consultations and faster disposition during the junior doctors' strike in 2020 in Korea.

## Figures and Tables

**Table 1:** Characteristics of patients who visited the emergency department during the strike and control periods.

	Strike period	Control period	*P* value
Total visits	1,121	1,496	
Male gender	582 (51.9)	782 (52.3)	0.888
Age (years)	56 (42–74)	55 (41–72)	0.188

Cause of visit			0.016
Disease	964 (86.0)	1229 (82.2)	
Injury^*∗*^	137 (12.2)	240 (16.0)	
Poisoning	11 (1.0)	21 (1.4)	
Other	9 (0.8)	6 (0.4)	

Route of visit			0.173
FD ambulances	374 (33.4)	457 (30.5)	
OPD	25 (2.2)	29 (1.9)	
Transferred	193 (17.2)	303 (20.3)	
Walk-in or other	529 (47.2)	707 (47.3)	

KTAS			<0.001
1	10 (0.9)	11 (0.7)	
2	94 (8.4)	121 (8.1)	
3	654 (58.8)	726 (48.7)	
4	297 (26.7)	526 (35.3)	
5	58 (5.2)	106 (7.1)	

Consciousness			0.376
Alert	1034 (92.2)	1401 (93.6)	
Verbal response	43 (3.8)	53 (3.5)	
Pain response	29 (2.6)	25 (1.7)	
Unresponsive	15 (1.3)	17 (1.1)	

Vitals signs			
Systolic BP (mmHg)	120 (110–140)	120 (110–140)	0.440
Diastolic BP (mmHg)	80 (70–90)	80 (70–90)	0.939
Heart rate (min^−1^)	86 (78–100)	84.5 (77–100)	0.087
Respiratory rate (min^−1^)	20 (20–21)	20 (20–22)	0.861
Body temperature (°C)	36.6 (36.5–37.2)	36.7 (36.5–37.1)	0.927
Pulse oxygen saturation	98 (96–98)	98 (97–99)	<0.001

Hospital admission	535 (47.8)	690 (46.2)	0.422
Mortality^*∗∗*^	26 (2.5)	35 (2.6)	0.891
EDLOS (min)	326 (123–318)	359 (147–391)	<0.001

^*∗*^Injury includes trauma, environmental injury, burns, and submersions. ^*∗∗*^Mortality includes mortality in the emergency department and after admission and expected death after discharge. Numbers in parentheses represent percentage or interquartile ranges. FD: fire department; OPD: outpatient department; KTAS: Korean Triage and Acuity Scale; BP: blood pressure; EDLOS: emergency department length of stay.

**Table 2:** Frequency of consultations (*P* < 0.001)

Number of consultations	Strike period (%) (*n* = 1,121)	Control period (%) (*n* = 1,496)
0	700 (62.4)	763 (51.0)
1	392 (35.0)	613 (41.0)
2	26 (2.3)	99 (6.6)
3	3 (0.3)	21 (1.4)

**Table 3:** Adjusted odds ratio for the effects of the doctors' strike, demographic, and clinical parameters on hospital mortality.

Variable	Odds ratio (95% confidence interval)	*P* value
Doctors' strike period	0.831 (0.472–1.444)	0.515
Male gender	1.415 (0.081–2.500)	0.224
Age	1.036 (1.017–1.058)	<0.001

Event		
Disease	1.0	
Injury	0.561 (0.089–1.931)	0.437
Poisoning	0.000 (0.000–22599)	0.983

KTAS	0.049 (0.298–0.805)	0.005
Consciousness (AVPU)^*∗*^	1.360 (1.062–1.721)	0.012
Systolic blood pressure^*∗*^	1.441 (1.144–1.806)	0.002
Heart rate^*∗*^	1.263 (0.917–1.719)	0.143
Respiratory rate^*∗*^	1.052 (0.819–1.348)	0.688
Body temperature^*∗*^	1.003 (6.130–1.558)	0.991
Pulse oximetry^*∗*^	1.346 (1.024–1.753)	0.030

^*∗*^Values were transformed into scores according to the National Early Warning Score before incorporated into the logistic regression model. KTAS: Korean Triage and Acuity Scale; AVPU: alert, verbal, pain, unresponsive.

## Data Availability

The data used to support the findings of this study have not been made available because of the relevant hospital policy.
